# Experience and perceptions of Social Prescribing interventions; a qualitative study with people with long-term conditions, link workers and health care providers

**DOI:** 10.12688/hrbopenres.13762.3

**Published:** 2024-01-09

**Authors:** Declan J. O'Sullivan, Lindsay M. Bearne, Janas M. Harrington, Joseph G. McVeigh

**Affiliations:** 1 Discipline of Physiotherapy, School of Clinical Therapies, College of Medicine and Health, University College Cork, Cork, T12X7OA, Ireland; 2 Population Health Sciences Research Institute, University of London, Cranmer Terrace London, England, SW17 0RE, UK; 3 School of Public Health, College of Medicine and Health, University College Cork, Cork, T12 XF62, Ireland

**Keywords:** Social Prescribing, long-term conditions, focus groups, link worker, community health worker, self-management

## Abstract

**Background:**

Long-term conditions (LTC) are a leading cause of reduced quality of life and early mortality. People with LTC are living longer with increasing economic and social needs. Novel patient centred care pathways are required to support traditional medical management of these patients. Social Prescribing (SP) has gained popularity as a non-medical approach to support patients with LTC and their unmet health needs. The current focus group study aims to explore the experiences and perceptions to SP interventions from the perspective of people with long-term conditions, link workers, healthcare providers and community-based services.

**Methods:**

Six toeight participants will be recruited into three specific 60 to 90 minute focus groups relative to their role as a patient, link worker and community-based service. 8 to12 participants with a Health care provider and GP background will be interviewed individually online. The participants within these focus groups and semi-structured interviews will be invited to provide opinions on what factors they think are important to the successful implementation of a SP service from their respective stakeholder positions. The data will be recorded and exported to NVivo software for further analysis using Thematic Reflexive analysis methods. Coded categorical data will inform emerging themes from which a narrative summary will be consolidated and presented for dissemination.

**Conclusion:**

The conclusions made from this study will help inform the next study, which will aim to develop a pilot SP service for patients with long-term musculoskeletal conditions as part of an overall larger project.

## Introduction

### Background and rationale

Long-term conditions (LTC) are typically characterised as non-self-limiting, persistent specific illness or multiple illnesses co-existing simultaneously ^1, 2^ which cause reduced quality of life and physical capacity and increase with age ^3^. Population growth, decreased socioeconomic circumstances and increasing age of individuals living with LTC place an extensive burden on society ^4^. Management of LTCs is challenging. Biomedical approaches targeting the underlying pathophysiology of the disease ^5^ have failed to address how an individual experiences ill-health ^6^. It is becoming evident that inadequacies in health provision to those in the middle to low socioeconomic groups requires evidence-based and cost-effective methods of managing patients with multifaceted LTC ^7– 9^. Self-management approaches ^10^ aiming to improve the day-to-day management of chronic illness include positive lifestyle changes and health literacy education ^11– 13^ to help reduce economic burden by empowering individuals to cope with their condition.

Social Prescribing (SP) is a self-management approach involving the utilisation of services already embedded in the community, and may act as short and long-term support for patients with health and wellbeing issues ^14^. SP emerged over a decade ago ^15^ and is growing across many countries to support those lower socioeconomic communities who have increased prevalence of long-term health and wellbeing conditions ^16^. SP involves the use of a lay person “link worker” or “community health worker” who facilitates the acquisition of tertiary non-clinical services after receiving a referral from a General Practitioner (GP) or other health care providers (HCP) ^17, 18^. SP models may be categorised as ‘broad’, involving light touch signposting to financial, employment or housing support services ^19^; referral to community groups (art/exercise therapy) ^14^ or more ‘tailored’ where support is more intensive to address unmet physical and mental health needs ^16, 20^. The evidence base around the use of SP models is emerging but of mixed results ^14, 21, 22^. Reviews have highlighted the lack of robust methodological evaluation ^22, 23^, inappropriate use of comparative outcome measures, high attrition rate and lack of appropriate controls ^14, 24^ as barriers to proper scientific evaluation. With so many different SP models each reflecting the community’s needs, it is difficult to establish what factors influence its successful implementation from the perspective and experiences of all the relevant stakeholders. The outcomes from this qualitative research will inform development of a later pilot study examining the effectiveness of broad or tailored SP models in people with long-term musculoskeletal conditions. 

***Aims and objectives.*** The aim of this qualitative study is to explore the implementation, utility and effectiveness of SP interventions in people with LTCs and those link workers, health care professionals and community based services who are engaged in SP interventions. The information collated from this study will inform a later pilot study examining the effectiveness of a specific SP intervention for individuals with long-term musculoskeletal conditions.

## Study design

### Design

This study will utilise qualitative methodology ^25^. The study will include four focus groups and one series of one-to-one interviews. One focus group will be conducted with people with LTC who have participated in SP programmes, one with link workers, one focus group will be conducted with HCPs who can refer to SP programmes and a focus group with community-based services that facilitate referrals from link workers. Each focus group will consist of six-to-eight participants. Individual semi-structured interviews will be conducted with 12 GP’s.

The four focus groups will be held on-line primarily but may also be in-person if online opportunities are unavailable. Each focus group discussion will be 60 to 90 minutes long. The semi-structured interviews with GPs will be held in person or Microsoft teams, each lasting 35 to 45 minutes depending on the time constraints of the GP. 

A predefined interview schedule will be developed and piloted with people with LTC and Link-workers to ensure important issues relevant to this population may be thoroughly explored through appropriate questions. The focus groups and semi-structured interviews will be moderated by Declan O’Sullivan (DOS) (principle researcher) and assisted by an experienced researcher in qualitative research (JMcV). This study will use a qualitative descriptive design ^26^ which will enable participants to voice their opinions and permit researchers to explore the phenomenon of interest ^27^. Further focus groups and interviews will be conducted if the information power has not been reached with the planned number of focus groups or interviews ^28^.

## Participants

### Sample

Four focus groups comprising of six-to-eight participants each and 12 one-to-one semi-structured interviews will be conducted. The sample size and information power required to address a study’s aim is difficult to estimate in advance of undertaking qualitative research ^28– 30^. To address this, guidance was taken from previous researchers who have examined this area, for example Malterud *et al.* ^28^ recommended that the aims of the study, sample specificity, use of established theory, quality of the dialogue and analysis strategy influence your sample size to achieve sufficient information power. In consideration of this work, it is believed that the narrowness of this study’s aim coupled with the study participants’ experience of the phenomenon of interest (SP) will enable extraction of all relevant information from our planned focus groups. The data being sought from the study participants is not complex but given the participant’s background is expected to be rich, which should facilitate a thorough analysis of data and identification of emerging thematic codes. The design of this study has committed to conducting 12 one-to-one semi-structured interviews based on the research conducted by Guest *et al.* ^30^ who reported that 97% of thematic codes could be identified with 12 interviews. In recognising, the necessity for systematic reflection and reviews throughout the data collection process, further focus groups or interviews will be conducted until thematic exhaustion has been reached.

### Inclusion criteria

The participants recruited for this study will be:

1. People living in middle-to-low socioeconomic areas within the South -South West Hospital group regions with LTCs who are currently or have in the past engaged with SP interventions (within the last six months). Participants will be over the age of eighteen years and be able to communicate in English (n=6–8)

2. Link workers embedded within a community who facilitate the implementation of a SP service on behalf of a voluntary or governmental agency (n=6–8)

3. Healthcare practitioners (Physiotherapists, Occupational therapists, Speech and language therapists, Social workers and GPs) who currently refer or previously referred patients directly to the link worker within a SP service (n=8–10)

4. Existing or past community-based service providers of SP interventions (n=6–8).

### Participant Exclusion criteria

1. Individuals who do not have a LTC or are under the age of 18. Those with existing psychiatric illness or insufficient English to enable informed consent to the study or where participation in the study may be detrimental to their health. 

### Ethical and regulatory considerations

Patient information sheets (Extended Data) will be provided and written informed consent (Appendix 5) will be acquired prior to and digitally recorded on the date of the focus group discussion. Participant confidentiality will be prioritised throughout the duration of this study. All participants’ data will be systematically anonymised digitally and only accessible by the principal researcher (DOS) and 1 ^st^ research supervisor (JMcV). The participants may withdraw from the study at any stage prior to and after the focus group discussion and their data will not be used in this study and deleted fully. This study will be submitted for ethical approval from University College Cork, Clinical Research Ethics Committee (CREC).

### Recruitment

Purposive sampling will be used to recruit participants voluntarily to take part in four focus groups and one semi-structured interview from regions identified as middle to low socioeconomic areas across the Health Service Executive, South-Southwest hospital group regions.

Purposive sampling will be used to recruit participants for each focus group and interview group:

Focus group one: Adults with long-term conditions (n=6–8) from diverse socioeconomic backgrounds

Focus group two: Healthcare providers including physiotherapist, occupational therapists, speech and language therapists, GPs, and social workers (n=8–12)

Focus group three: A ‘Gate Keeper’ will be used to recruit link workers (n=6–8) for a focus group.

Focus group four: Facilitators working in community-based services (n=6–8)

Interveiw group: General practitioners

To recruit participants for group one, group three and group four focus groups, a formal introductory email (Extended data) will be forwarded to the manager (Gate Keeper) of SP interventions in the South-South West Hospital Group (SSWHG) in Munster. This email will inform them of the details of the study, how many participants are required, and a request to share information about participation with their staff (link workers), patients and community-based SP services as appropriate ( *e.g. via* word of mouth, telephone or email). Participants for this group will also be recruited through flyers, social media posts and posters made by the research team and placed in community resource centres, General Practitioner (GP) practices and community-based services already providing SP interventions.

Participants for group two focus group will be recruited with a formal introductory email (Extended data) to the managers (Gate Keepers) of local physiotherapy, occupational therapy, speech and language therapy and social worker departments outlining details of the study, how many participants are required, and a request to share information about participation with their staff. Group 2 may also be recruited indirectly through flyers, social media posts and posters containing the contact details of the principle researcher (DOS).

For the interview group, GP’s will be recruited through a formal introductory email (Extended data) to local clinics outlining why GPs are being recruited, details of the study, the duration of the interview and how the interview will be conducted.

Groups two and four may also be recruited indirectly through flyers, social media posts and posters containing the contact details of the principal researcher (DOS).

Interested participants can contact the principal researcher (DOS) who will arrange a time to contact them by telephone. At that initial contact, DOS will explain the study, answer any questions, and screen the participant against the inclusion and exclusion criteria. DOS will provide potential participants a plain language participant information leaflet Extended data) with relevant information about the study and a consent form (Extended data).

### Focus group procedure

All participants will be provided with a written information sheet and written consent form prior to commencement of the focus group discussion. The participants will be sent an email with a M-Teams meeting link one month prior to the focus group and a reminder one week in advance clearly indicating the date and time for the discussion. An interview schedule will be formulated by principle researcher (DOS), reviewed by JMcV and piloted prior to the focus group discussion. The facilitator (DOS) will provide at the beginning of each focus group a brief summary/reminder of the purpose of the study and focus group, and outline focus group etiquette and conduct rules. The facilitator will then ask participants to introduce themselves very briefly. There will be eight to 14 open ended questions designed to encourage engagement based around sub-themes of; attitudes and expectations; experience of SP; impact of SP; recommendations and finally exit questions to determine if there were any outstanding or new themes that had not been explored. Participants will be reimbursed for any financial cost associated with travel to and from the focus group if they are held in person. If the focus groups are held in person, a suitable venue will be identified with appropriate wheelchair access. The discussion will be preceded by a quick synopsis of the aims and objectives of the study and moderated by DOS in its entirety and guided by the interview guide. A non-participant mediator (JMcV) will track the questions and ensure all topics are followed up.

### Interview procedure

All participants will be provided with a written information sheet and written consent form prior to commencement of the interviews. The participants will be forwarded a M-Teams meeting link by email for the date and time of the interview one month prior to the interview and an email reminder one week prior to it. An interview schedule will be formulated by DOS, reviewed by JMcV and piloted prior to the interview data collection. The interview will be recorded once consent has been achieved.

### Data collection and analysis

Qualitative data will be collected *via* the focus groups and interviews. Field notes will be taken. Focus groups will be audio recorded using an Olympus Digital Voice Recorder WS-853 or equivalent. Audio-recordings will be immediately transferred to the secure OneDrive folder and deleted from the recording device. The audio files will be transcribed anonymously by the principle researcher (DOS) with all identifiers removed and replaced with an ID number through an on-line proprietary software transcribing software (Otter.ai) ^31^ or open access otranscribe ^32^. If the focus groups or one to one interviews are conducted on line with Microsoft Teams ^33^, an automated transcribing application embedded within this software will perform the transcription. The data will be exported to a proprietary software NVivo ^34^ or open access Aquad ^35^ which is username and password protected software used for the analysis of unstructured text as found in a group discussion ^34, 36^. The NVivo software will be stored on the principal researcher’s (DOS) laptop, which is, also pin code protected. A further encrypted pin will be required to access the data file when using the NVivo software. 

A qualitative descriptive design will be utilised for the analysis of the data ^27^. The data will be scrutinised qualitatively (DOS) using Reflexive Thematic analysis framework ^37, 38^ to ensure transparency and reduce bias ^39^. Using an inductive approach, the following six-step methodology will be employed; data familiarisation; data coding; generation of initial themes; developing and reviewing of themes; redefining, defining and naming of themes and finally write up ^38^ to enable ‘illustrative quotations’ ^40^ to consolidate narrative conclusions ^41^. The final phase of the thematic analysis will involve interpretation and reporting of findings. As a quality check, member checking of the dataset may be implemented if after data analysis our interpretation of the data may potentially be recognisable to some participants ^42^. If this occurs, participants will be asked to read and comment on the analysis as to its accuracy of their experiences

### Data management

Recorded audio files will be saved only until transcription is complete, at which time they will be deleted by the principal researcher DOS. Transcripts will be stored in a secure folder on the project OneDrive. We will store any paper consent forms until such time as they can be scanned and stored electronically, expecting that many consent forms will be scanned and returned by email by the participants. Paper consent forms will then be shredded. We will store participant’s consent information (Extended data) on a secure Excel file on the project OneDrive folder. 

The electronic data will be stored on an encrypted file on the UCC SharePoint in line with the University’s Code of Research Conduct Version 2.3.2019. The qualitative data will be stored in a .sav file on the UCC secure server (OneDrive) on the personal work computers of the principal researcher (Declan O’ Sullivan) and PhD supervisor Dr J. McVeigh. We do not anticipate storing any physical data, as consent forms will be shredded and saved electronically.

The electronic data will be stored in line with the Universities Code of Research conduct, for at least 15 years after the publication of any reports or papers arising from the study, after which time the principal researcher DOS will destroy the data.

This electronic dataset will remain within the School of Clinical Therapies and will not be made publicly available for open data sharing purposes. Participants in the focus groups will not be asked for their consent to share their data publicly. The analysed anonymous data will likely be presented as project output in dissemination such as conferences, journal submissions, and will be included in the final report. Declan O Sullivan: the principal researcher will be responsible for storing and protecting the data collected. Access to this data will be restricted to the research team. Generated data associated with this research will be stored for a minimum of ten years according to university regulations and the data will not be reused. A PDF of the final version of this study will be forwarded to the participants of the focus group by email or post and will simultaneously be submitted to an Open Access journal for publication and dissemination.

### Definition of end of study

The focus group will terminate when all participants have departed from the focus group venue. This study will end when the final version of the study has been approved by the research team and it has been submitted to an appropriate journal for publishing.

### Quality assurance procedures

The quality of this study will be underpinned by ensuring there is clarity of purpose; recruiting appropriate participants; skilful moderator with effective questions; detailed and systematic analysis of the data ^43^.

### Protocol study status

This study is awaiting ethics approval

#### Expenses and benefits

Participants of the focus group discussion will be remunerated for the cost of travel to and from the venue hosting the focus group discussion only and will not be remunerated if the focus groups are held online. Participants within the interview group (GPs) will not be remunerated for their time.

#### Insurance

All research involving patients/volunteers must be approved by UCC Sponsor's Office before study commencement date. When this approval is granted all participants of this study will be indemnified in accordance with the terms and conditions of the UCC policy.

## Data Availability

Figshare: Experience and Perceptions of Social Prescribing interventions; a Qualitative study with people with Long-term conditions, Link workers and Health care providers. O’Sullivan *et al.* https://doi.org/10.6084/m9.figshare.23527983 This project contains the following extended data: Appendix 1 Participant Information Sheet-Patient Appendix 2 Participant Information Sheet-Link Worker Appendix 3 Participant Information Sheet-Healthcare Provider Appendix 4 Participant Information Sheet-Community based service Appendix 5 Consent form Appendix 6 Email to 'gatekeeper'-link worker Appendix 7 Email to 'gatekeeper'-Healthcare provider Appendix 8 Email to GP clinics Appendix 9 Interview guide Appendix 10 Protocol Amendment History Data are available under the terms of the Creative Commons Attribution 4.0 International (CC BY 4.0)
