# Supplementary material for: Experience and perceptions of Social Prescribing interventions; a qualitative study with people with long-term conditions, link workers and health care providers
Source: HRB Open Res. 2024 Jan 9;6:42. Originally published 2023 Sep 1. [Version 3] doi: 10.12688/hrbopenres.13762.3 (PMC10822040; doi:10.12688/hrbopenres.13762.3)
Supplement: Peer review history

# Reviewer report: Reviewer response for version 3

**DOI:** 10.21956/hrbopenres.15155.r37722

**Published:** 2024-01-27

**Authors:** Fiona Cramp

**Affiliations:** 1 School of Health and Social Wellbeing, University of the West of England, Bristol, UK

The authors have addressed all of my previous comments and I have nothing further to add.

Is the study design appropriate for the research question?

Yes

Is the rationale for, and objectives of, the study clearly described?

Yes

Are sufficient details of the methods provided to allow replication by others?

Partly

Are the datasets clearly presented in a useable and accessible format?

Not applicable

Reviewer Expertise:

Long term conditions, physical activity, complex interventions

I confirm that I have read this submission and believe that I have an appropriate level of expertise to confirm that it is of an acceptable scientific standard.

# Reviewer report: Reviewer response for version 2

**DOI:** 10.21956/hrbopenres.15118.r36543

**Published:** 2023-10-18

**Authors:** Candice Oster

**Affiliations:** 1 Caring Futures Institute, College of Nursing and Health Sciences, Flinders University, Adelaide, SA, Australia

Thank you for your amendments. I have not further comments.

Is the study design appropriate for the research question?

Yes

Is the rationale for, and objectives of, the study clearly described?

Partly

Are sufficient details of the methods provided to allow replication by others?

Partly

Are the datasets clearly presented in a useable and accessible format?

Not applicable

Reviewer Expertise:

Social prescribing, qualitative research, chronic condition management.

I confirm that I have read this submission and believe that I have an appropriate level of expertise to confirm that it is of an acceptable scientific standard.

# Reviewer report: Reviewer response for version 1

**DOI:** 10.21956/hrbopenres.15054.r36023

**Published:** 2023-10-06

**Authors:** Fiona Cramp

**Affiliations:** 1 School of Health and Social Wellbeing, University of the West of England, Bristol, UK

First line of the abstract Methods - change 'Six-eight...' to 'Six to eight...' and 'link worker and community-based service' to 'link worker or community-based service'. Sentence two, change to 'Eight to12 health care providers and GPs will be interviewed individually online.'  

 Background and rationale

 First sentence: '...and increases with age ^3^' should read 'and increase with age ^3^'. 

 'Management of LTCs are challenging'  should be 'Management of LTCs is challenging'.

 Overall, the background sets the scene for the study by identifying the limited evidence base and wide range of models of social prescribing.  It would be useful to include a definition of self-management towards the beginning of the background.

 The aim of the research is clear and appropriate.  It is clear that this research is needed and overall methods are appropriate.

 Design:  From paragraph one it is not entirely clear how many focus groups are planned as initially it is stated that there will be three focus groups and subsequently there is a statement that focus groups will be continued until sufficient information power has been reached.  This needs further clarification.  A sentence also needs to be added to justify the difference data collection methods for each stakeholder group.  The abstract states that the focus groups will be 60 minutes but the main text indicates 60-90 minutes, this needs to be consistent.  

 Sample: It is not clear how the proposed overall sample of thirty has been reached.  How do you know that information power will be achieved with this number?  The sentence relating to numbers per focus group should appear under the sample rather that in the recruitment section: 'We aim to have 6–8 participants per focus group and eight-12 semi-structured interviews.'

 The background indicated that the middle to low socioeconomic groups were those with greatest support needs.  How will you ensure that there is adequate representation from this population within your sample?  

 It needs to be made clear that the 'exclusion criteria' are specific to the patient population.

 Focus group procedure - Reword the following sentence:  'The participants will be forwarded a M-Teams ^31^ meeting link by email for the date and time of the focus group one month prior to the focus group discussion by email and an email reminder one week prior to it.'   Suggestion:  'The participants will be sent an email with a M-Teams ^31^ meeting link one month prior to the focus group and a reminder one week in advance clearly indicating the date and time for the discussion.'

 Will the moderator be present during the focus groups?

 How will you decide whether to conduct the focus group via Microsoft Teams or in person?

 Data collection and analysis and data management sections are clear. 

 The paper could be enhanced with consideration of the researchers perspective and how the data that is collected will subsequently be used.

Is the study design appropriate for the research question?

Yes

Is the rationale for, and objectives of, the study clearly described?

Yes

Are sufficient details of the methods provided to allow replication by others?

Partly

Are the datasets clearly presented in a useable and accessible format?

Not applicable

Reviewer Expertise:

Physiotherapy, long term conditions, complex interventions

I confirm that I have read this submission and believe that I have an appropriate level of expertise to confirm that it is of an acceptable scientific standard, however I have significant reservations, as outlined above.

# Response

**Published:** 2023-11-10

**Authors:** Declan J. O Sullivan

**Affiliations:** Discipline of Physiotherapy, Univeristy College Cork, Cork, Cork, Ireland

19 ^th^ Oct 2023,

 Re: Experience and perceptions of Social Prescribing interventions; a qualitative study with people with long-term conditions, link workers and health care providers.

 Authors:  O’Sullivan D1, Bearne LM.2, Harrington JM.3, McVeigh JG1

 Dear Prof Cramp,

 Many thanks for reviewing my protocol and for your helpful comments and advice. Please see below a point by point response to your comments.  The relevant text changes are highlighted in the main study protocol.

 Kind regards

 Declan O’Sullivan

**Response to reviewer**

 1. First line of the abstract Methods - change 'Six-eight...' to 'Six to eight...' and 'link worker and community-based service' to 'link worker or community-based service'..' 

**Response:** Thank you; I have changed this to reflect your recommendations (Line 25)

 2. Sentence two, change to 'Eight to 12 health care providers and GPs will be interviewed individually online

**Response:** Thank you; I have changed this to reflect your recommendations (Line 26)

 3. Background and rationale

 First sentence: '...and increases with age (3)' should read 'and increase with age (3)'.

**Response:** Thank you I have changed this to reflect your recommendations (Line 47)

 4. 'Management of LTCs are challenging’ should be 'Management of LTCs is challenging'.

**Response:** Thank you I have changed this to reflect your recommendations (Line 49)

 5. Overall, the background sets the scene for the study by identifying the limited evidence base and wide range of models of social prescribing.  It would be useful to include a definition of self-management towards the beginning of the background.

**Response:** Thank you I have changed this to reflect your recommendations (Line 52-55)

 6. Design:  From paragraph one it is not entirely clear how many focus groups are planned as initially it is stated that there will be three focus groups and subsequently there is a statement that focus groups will be continued until sufficient information power has been reached.  This needs further clarification.  A sentence also needs to be added to justify the difference data collection methods for each stakeholder group. 

**Response:** Thank you for your point. I have amended the text and added some clarification,  hopefully it  is much clearer for the reader (line 84-102).

 7. The abstract states that the focus groups will be 60 minutes but the main text indicates 60-90 minutes, this needs to be consistent. 

**Response:** Thank you. I can see that now. I have changed the text accordingly (line 25)

 8. Sample: It is not clear how the proposed overall sample of thirty has been reached.  How do you know that information power will be achieved with this number? 

**Response:** Thank you for your point. I am aware of the difficulties in identifying the correct sample size to validate our findings. I am aware of what factors might influence the arrival at an optimal sample size and the necessity to extract all thematic codes before deciding if more participants are required to reach information power. I think I have addressed your concerns in lines 105-120

 9. The sentence relating to numbers per focus group should appear under the sample rather that in the recruitment section: 'We aim to have 6–8 participants per focus group and eight-12 semi-structured interviews.'

**Response:** Thank you. I have amended this in text (line 106)

 10. The background indicated that the middle to low socioeconomic groups were those with greatest support needs.  How will you ensure that there is adequate representation from this population within your sample? 

**Response:** Thank you for your comment. I have highlighted in text that I will use purposive sampling to recruit suitable participants from existing SP services and GP clinics and health resource centres from middle to low socioeconomic areas within the South-Southwest hospital group regions (line 154-156).  I have also highlighted this in the inclusion criteria (Line 124).

 11. It needs to be made clear that the 'exclusion criteria' are specific to the patient population.

**Response: **Thank you for your point. I have included this information in text now (line 138).

 12. Focus group procedure - Reword the following sentence:  'The participants will be forwarded a M-Teams31 meeting link by email for the date and time of the focus group one month prior to the focus group discussion by email and an email reminder one week prior to it.'   Suggestion:  'The participants will be sent an email with a M-Teams31 meeting link one month prior to the focus group and a reminder one week in advance clearly indicating the date and time for the discussion.'

**Response:** I can see this does require some grammar changes and thank you for your suggestion. I have changed this within the text (line 193-195)

 13. Will the moderator be present during the focus groups?

**Response:** Yes the moderator will be present (line 197).

 14. How will you decide whether to conduct the focus group via Microsoft Teams or in person?

**Response:** The recruitment sample, availability of the participants to travel to a suitable venue and finally a COVID resurgence with governmental restrictions will decide whether the focus groups will be held in person or via M-Teams.

 15. The paper could be enhanced with consideration of the researcher’s perspective and how the data that is collected will subsequently be used.

**Response:** Thank you again for your advice. I feel I have addressed this point sufficiently in response to reviewer 1 comments. I have reworded the abstract (line 35 to 37) and background (Lines 71-73) to convey how the data from this study will influence a later study examining the effectiveness of a tailored SP model to MSK long-term conditions

# Reviewer report: Reviewer response for version 1

**DOI:** 10.21956/hrbopenres.15054.r36018

**Published:** 2023-09-15

**Authors:** Candice Oster

**Affiliations:** 1 Caring Futures Institute, College of Nursing and Health Sciences, Flinders University, Adelaide, SA, Australia

This article is a protocol for a qualitative study exploring the experiences and perceptions of social prescribing of people with long-term conditions, link workers and health care providers. Participants will be those receiving or delivering social prescribing interventions (social prescribing participants, health care providers, General Practitioners (GPs), and community-based services). Data will be collected via semi-structured focus groups (with social prescribing participants with long-term conditions, link workers, and community-based services) and one-to-one interviews (with health care providers and GPs). Data will be analysed using reflexive thematic analysis. Study conclusions will inform the development of a pilot social prescribing service for people with long-term conditions.

 The rational for the study is described. In particular, the authors discuss heterogeneity in social prescribing models and lack of clarity around what factors influence the success of models of social prescribing. Some further discussion is warranted here to link the rationale to the use of outcomes to inform development of a pilot program, as stated in the abstract. It is not clear why there is a need for a pilot program given that people with long-term conditions are already being provided social prescribing (i.e., you are recruiting people with long-term conditions who have received social prescribing). Some contextual information on existing programs and their inclusion of people with long-term conditions, and potentially the need to tailor social prescribing to particular population groups (if that is the rationale for the study), would be useful.

 The study aim is quite broad in focusing on experiences and perceptions of social prescribing. Are you looking to explore specific elements that relate to the utility/effectiveness of social prescribing for people with long-term conditions (e.g., barriers and enablers)? It might be worth including some objectives that relate to the study population and the ultimate goal of outcomes being used to inform the development of the new service. 

 Methods: It would be good to clarify early on in the methods section that there will be one focus group conducted for each of the three types of participants with 6-8 participants in each focus group. In addition, some clarity around how you will determine when sufficient information power is reached would be useful, particularly given the plan to conduct one focus group per participant group (i.e., how is information power determined and whether further focus groups will be conducted if this threshold isn’t reached with the planned number of focus groups).

 Under Recruitment, could the ‘Groups’ be more clearly delineated in terms of which will take part in focus groups versus interview? E.g., ‘Group one (focus group)’, Group two (interviews)’ etc? Otherwise, it reads as though there are four focus groups. It is also stated that *‘Participants for group two focus group will be recruited …*’ – aren’t these participants to be interviewed individually? 

 Interview procedure: Will the interviews cover the same broad domains that were stated for focus groups?

 Data collection and analysis: There is some repetition here with the previous section, e.g., around audio recording (which is mentioned under interview procedure) and the use of a semi-structured interview guide. Who will undertake the thematic analysis? How will member checking be undertaken and what is the rational for this? Also, the use of the analysis to inform development of a pilot social prescribing service should be stated in the main text of the article in addition to in the abstract.

 Definition of end of study: Is it necessary to mention the end of the focus groups here?

 Expenses: Will interview participants be remunerated?

Is the study design appropriate for the research question?

Yes

Is the rationale for, and objectives of, the study clearly described?

Partly

Are sufficient details of the methods provided to allow replication by others?

Partly

Are the datasets clearly presented in a useable and accessible format?

Not applicable

Reviewer Expertise:

Social prescribing, qualitative research, chronic condition management.

I confirm that I have read this submission and believe that I have an appropriate level of expertise to confirm that it is of an acceptable scientific standard, however I have significant reservations, as outlined above.

# Response

**Published:** 2023-09-26

**Authors:** Declan J. O Sullivan

**Affiliations:** Discipline of Physiotherapy, Univeristy College Cork, Cork, Cork, Ireland

18 ^th^ Sept 2023,

 Re: Experience and perceptions of Social Prescribing interventions; a qualitative study with people with long-term conditions, link workers and health care providers.

 Authors:  O’Sullivan D1, Bearne LM.2, Harrington JM.3, McVeigh JG1

 Dear Dr Oster,

 Many thanks for reviewing my protocol. Thank you for your comments and advice. I have extracted comments from your report that require actions. I will address each of these below. The relevant text changes are highlighted in the main study protocol.

 Kind regards

 Declan O Sullivan

**Response to reviewer**

 1. The rationale for the study is described. In particular, the authors discuss heterogeneity in social prescribing models and lack of clarity around what factors influence the success of models of social prescribing. Some further discussion is warranted here to link the rationale to the use of outcomes to inform the development of a pilot program, as stated in the abstract.

**Response: **Thank you for your comments. I agree this needs clarification.

**Action:** I have amended the final sentence in the background and rationale paragraph to address this ambiguity (line 69)

 2. It is not clear why there is a need for a pilot program given that people with long-term conditions are already being provided social prescribing (i.e., you are recruiting people with long-term conditions who have received social prescribing).

**Response:** My Ph.D. is exploring the effectiveness of Social Prescribing in the management of long-term musculoskeletal conditions specifically.  This FG may inform the optimal SP methodology for individuals with these conditions.

**Action:** I have reworded the abstract conclusion to reflect this. (Line 34)

 3. Some contextual information on existing programs and their inclusion of people with long-term conditions, and potentially the need to tailor social prescribing to particular population groups (if that is the rationale for the study), would be useful.

**Response: **I have revised the text and I agree that this needs stronger emphasis.

**Action: **I have replaced ‘specific’ to ‘tailored’ and I have made the point that evidence surrounding the use of these SP models is mixed. I have also highlighted that the next study will be examining the effectiveness of each model in long-term musculoskeletal conditions. (line 63)

 4. The study aim is quite broad in focusing on experiences and perceptions of social prescribing. Are you looking to explore specific elements that relate to the utility/effectiveness of social prescribing for people with long-term conditions (e.g., barriers and enablers)? It might be worth including some objectives that relate to the study population and the ultimate goal of outcomes being used to inform the development of the new service.

**Response:** Thank you for your comment. I have looked at my aim and I have elaborated more on it to give the reader a full sense of what I am trying to achieve with this study.

**Action:** I have changed the wording of the “Aims and Objectives” paragraph to reflect this. (line 74)

 4. Methods: It would be good to clarify early on in the methods section that there will be one focus group conducted for each of the three types of participants with 6-8 participants in each focus group.

**Response:** I can see the potential for confusion with this and I have amended it to reflect your advice

**Action:** I have added a sentence to the paragraph under “Design” to add clarity to the design process. (line 84)

 5. In addition, some clarity around how you will determine when sufficient information power is reached would be useful, particularly given the plan to conduct one focus group per participant group (i.e., how is information power determined and whether further focus groups will be conducted if this threshold isn’t reached with the planned number of focus groups).

**Response:** Thank you for your advice. I have highlighted this point more in the design.

**Action:** I have reworded the sentence relating to information power to highlight the point that more focus groups will be conducted until sufficient information power has been reached. (line 91)

 6. Under Recruitment, could the ‘Groups’ be more clearly delineated in terms of which will take part in focus groups versus interviews? E.g., ‘Group one (focus group)’, Group two (interviews)’ etc? Otherwise, it reads as though there are four focus groups.

**Response:** Thank you for highlighting this. For this study, I will conduct four focus groups (Gp 1 Patients, Gp 2 Referrers, Gp3 Link workers, Gp4 Community based services, and Interview Gp General practitioners)

**Action:** I have added more information to the paragraph under “recruitment” to reflect this need for clarity.  I have added labels to the groupings of participants. (lines 135-149)

 7. It is also stated that ‘Participants for group two focus group will be recruited …’ – aren’t these participants to be interviewed individually?

**Response: **Focus group 2 will not be interviewed individually. Focus group 2 will contain health care practitioners including physiotherapists, occupational therapists, speech and language therapists and social workers. They will be recruited through direct email to gatekeepers and through social media.

**Action:** In actioning your previous point I hope this will be more clear to the reader now.  I have also re-structured the paragraph slightly to bring make this more concise. (line 159-164)

 8. Interview procedure: Will the interviews cover the same broad domains that were stated for focus groups?

**Response:** Thank you for your question. Yes, the interviews will contain the same broad domains as stated for the focus group. This is highlighted in the paragraph under “interview Procedure”

**Action:** none is required at this time.

 9. Data collection and analysis: There is some repetition here with the previous section, e.g., around audio recording (which is mentioned under interview procedure) and the use of a semi-structured interview guide.

**Response:** Thank you for highlighting the repetition.

**Action:** I have deleted any further reference to audio recording processes and interview schedules within the “Data Collection and Analysis” paragraph. (line 206-227)

 10. Who will undertake the thematic analysis?

**Response:** Thank you, this was not clear within the text.

**Action**: I have added my initials to this sentence to inform the reader who was responsible for this task. (line 218)

 11. How will member checking be undertaken and what is the rationale for this?

**Response:** Thank you for highlighting this. Member checking is an important quality appraisal tool that may be utilized but not necessary depending on the outcome of the dataset analysis. 

**Action: **Under the “Data collection and Analysis” paragraph, I have rewritten this sentence to capture the rationale and procedure for member checking in the event it will be necessary. (line 224)

 12.  The use of the analysis to inform the development of a pilot social prescribing service should be stated in the main text of the article in addition to in the abstract.

 Response: Thank you very much for this advice. I agree this should have been highlighted more.

**Action:** I have taken this on board and made appropriate changes to the abstract and in-text in the final line of the “background and rationale” paragraph. (Line 34, Line 69)

 13. Definition of end of study: Is it necessary to mention the end of the focus groups here?

**Response:** As a novice researcher, I was following previous studies' headings.

**Action:** I have deleted this paragraph from the text.

 14. Expenses: Will interview participants be remunerated?

**Response:** Thank you for your comment. No, interview participants will not be reimbursed. Interviews will be conducted online and at a time convenient to them.

**Action:** This was clarified in the text. (Line 259-261)

 15. Is the rationale for, and objectives of, the study clearly described?

 Partly

**Response**: I hope with the advice and recommendations from the reviewer that the rationale and objectives are more clearly defined.

 16.  Are sufficient details of the methods provided to allow replication by others?

 Partly

**Response: **I have made the necessary changes to the text as proposed by the reviewer. I hope this will address any ambiguity to the reader.
